# Zinc oxide nanoparticles-doped curcumin-assisted recovery of rheumatoid arthritis and antioxidant status in experimental rabbits

**DOI:** 10.37796/2211-8039.1446

**Published:** 2024-06-01

**Authors:** Shanza Azeez, Mishal Fatima, Ouz Gul, Huzaifa Rehman, Muhammad A. Shad, Haq Nawaz

**Affiliations:** a Department of Biochemistry, Bahauddin Zakariya University, Multan, Pakistan; b Department of Medical Sciences, Times Institute Multan, Multan, Pakistan

**Keywords:** Antioxidant status, Complete Freund’s adjuvant, Oxidative stress, Rheumatoid factor, Rheumatoid arthritis, Zinc oxide nanoparticles-doped curcumin

## Abstract

**Introduction:**

Rheumatoid arthritis (RA) is a chronic autoimmune disease characterized by inflammation and synovial joint destruction.

**Aims:**

The current study investigated the possible beneficial effect of zinc oxide nanoparticles doped curcumin (ZnONPs-DC) on the recovery of RA and antioxidant status of experimental rabbits.

**Methods:**

RA was induced in experimental rabbits by injecting complete Freund’s adjuvant and collagen type-II emulsion (100 μL/kg body weight) in the base of their tail. Arthritic rabbits were orally treated with ZnONPs, curcumin, and ZnONPs-DC(250 μL/kg bodyweight). Serumsamples fromthe control and study groupswere collected before and afterRAinduction and after treatment. The sera were subjected to analysis of biological markers of RA and antioxidant status.

**Results:**

The complete Freund’s adjuvant and collagen type II treatment resulted in positive rheumatoid factor and C-reactive protein elevated oxidative stress and decreased antioxidant potential. Each treatment showed the absence of rheumatoid factor and C-reactive protein decreased oxidative stress and improved antioxidant potential compared to the control. However, ZnONPs-DC treatment showed a comparatively higher decline in serum malondialdehyde MDA content and an elevation in the antioxidant activity of RA animals.

**Conclusions:**

In conclusion, using zinc oxide nanoparticles-doped curcumin may be an effective anti-arthritic and anti-inflammatory drug in controlling RA.

## 1. Introduction

Rheumatoid arthritis (RA) is the most common chronic inflammatory condition, with symptoms including synovial joint hyperplasia, cytokine and chemokine production, joint inflammation, an autoimmune system such as rheumatoid factor (RF), and common conditions such as cardiovascular illness, psychological disorders, and bone erosion [1]. The initial hypothesis on the pathophysiology of RA pivoted to autoimmunity [2]. Numerous autoantibodies, including anti-citrullinated protein antibodies and RF, have been linked to RA [3]. In rheumatoid synovitis, oxidative stress produced within an inflamed joint can cause autoimmune reactions and synovial tissue damage. The promoters and effectors of articular damage are reactive nitrogen and reactive oxygen species (ROS) [4]. A five-fold increase in mitochondrial ROS production in whole blood and monocytes of RA patients was observed suggesting oxidative stress a pathogenic hallmark in RA [5]. RA is thought to affect 0.5–1% of mature individuals in advanced phases. The disease is more likely to affect women and older people than men. Muscle discomfort, inflammation, exhaustion, fever, appetite loss, weight loss, and an increase in localized bone loss around swollen joints are the common symptoms of RA [6].

With the ultimate goal of reaching remission, the therapy of rheumatic patients seeks to reduce inflammation and discomfort. The treatment is challenging because it involves nonpharmacologic strategies and many drug classes administered via various routes. Glucocorticoids (GC) are frequently used to reduce inflammation and ease pain. In the long run, disease-modifying anti-rheumatic drugs (DMARDs) are needed to spare GC and manage inflammation [7]. In RA patients, DMARDs significantly reduce the signs of illness and prevent the spread of the disease [8]. Due to low effectiveness, adverse reactions, and high costs of DMARDs, researchers are still exploring phytochemical-based novel therapies. Plant derived antioxidant compounds such as flavonoids, terpenes, quinones, catechins, alkaloids, and anthocyanins can influence the expression of pro-inflammatory signals. These compounds may be helpful in the treatment of RA. However, the poor physicochemical properties of pharmaceuticals obtained from natural sources, which are connected to their poor pharmacokinetics and bioavailability in the human body, reduce the therapeutic efficiency of these drugs [9]. Packaging bioactive compounds in a nanocarrier allows targeted drug delivery to the joints. Additionally, nanocarrier systems may improve the efficiency of some medications by increasing their solubility and protecting them from degradation in the bloodstream [10].

Curcumin is a polyphenol derived from *Curcuma longa* Linn’s rhizomes. The studies have demonstrated the therapeutic effects of curcumin on clinical and inflammatory parameters and identified several mechanisms involved in the onset of RA [11]. However, a unique delivery mechanism is needed to get curcumin to the site of action because its physicochemical qualities have been damaged, restricting its bioavailability. Researchers examined readily accessible solid lipid nanoparticles for treating arthritic patients to get around these restrictions. Contrary to free curcumin treatment, curcumin-bound solid lipid nanoparticle treatment dramatically improved disease markers and prevented imaging impairments in joints of arthritic rats [12]. Compared to regularly taking traditional medications, polymeric nanoparticle therapy showed much potential for enhancing treatment outcomes [13].

Zinc oxide nanoparticles (ZnO NPs), one of the most significant metal oxide nanoparticles, are currently used in many biological applications due to their suitability for natural use, cost-effectiveness, and acceptable safety profile [14]. Curcumin has exhibited effectiveness in treating RA when combined with various nanocarrier systems; however, no studies have been written on the therapeutic effects of zinc oxide nanoparticles-doped curcumin (ZnONPs-DC) on RA. Therefore, this study aimed to evaluate the impact of ZnONPs-DC on the antioxidant status of experimentally induced RA rabbits and its efficacy in treating RA. The study may be valuable contribution to the literature regarding the oxidant-antioxidant balance and treatment options for RA patients.

## 2. Materials and methods chemicals and reagents

Ethanol, Ammonium thiocyanate, Methanol, Iron chloride, Hydrogen peroxide, hypoxanthine, sodium phosphate, Complete Freunds Adjuvant, and sodium carbonate were purchased from Sigma Aldrich. Zinc nitrate hexahydrate and sodium hydroxide were purchased from Duksan. Ammonium Molybdate, Hydrochloric acid, Sulphuric Acid, Acetic Acid, Malondialdehyde, and butylated hydroxytoluene were purchased from British Drug Houses Chemical Ltd. Curcumin was bought from local Multan suppliers. 2, 2-diphenyl-1picrylhydrazyl (DPPH) was bought from FLUKA. All the chemicals were used without any further purification.

## 3. Study design

The present study evaluated the effect of ZnONPs-DC on recovery of RA, oxidative stress and antioxidant status in experimentally-induced RA rabbits using a randomized control design (RCD). The Departmental Research Ethical Committee, Bahauddin Zakariya University, Multan, Pakistan, approved the study (Approval No. Biochem/0-118/2023). The study was performed in three phases: 1) synthesis of ZnONPs and its doping on commercially available curcumin to synthesize ZnONPs-DC, 2) induction of RA in four groups of white New Zealand albino rabbits by injecting an emulsion of commercially available complete Freunds adjuvant and Collagen type II extracted from chicken feet skin, and 3) treatment of three groups of experimentally-induced RA animals with curcumin, ZnONPs, and ZnONPs-DC. The blood samples were taken before and after RA induction and treatment, and the sera were analyzed for RA biomarkers and parameters of oxidative stress and antioxidant potential. The data were analyzed by one-way analysis of variance (ANOVA), and the results were compared using Tukey’s multiple range test (SPSS version 19).

## 4. Nanoparticles synthesis

ZnONPs were synthesized utilizing the co-precipitation process [15]. 0.4M NaOH solution (100 ml) was gradually added to 0.2M zinc nitrate solution (100 ml), and stirred at 60 °C for two hours. The white precipitate obtained after filtration was washed with deionized water, dried at 75 °C in an oven for six hours, and ground to a fine powder. The ZnONPs-DC was synthesized by mixing 0.1 M ZnONPs suspension with 0.1M ethanolic curcumin solution, followed by stirring at 60 °C for two hours. The mixture was filtered, and the ZnONPs-DC was washed with deionized water, dried in an oven at 75 °C for six hours, and stored in an air-tight glass jar.

## 5. Physical characterization of nanoparticles SEM analysis

The morphology, size distribution, and surface nitrogen adsorption patterns of ZnONPs and ZnONPs-DC were determined by scanning electron microscopy (SEM), dynamic light scattering (DLS), and Brunauer-Emmet-Teller (BET) surface analysis techniques, respectively. The SEM, DLS, and BET analysis were performed using standard protocols on a Scanning electron microscope, Zeta Malvern nano-Zetasizer, Anton Par, and BELSORP mini X sorption analyzer, respectively [16–18].

## 6. Collagen extraction

The chicken feet were cleaned with distilled water, boiled for 30 min, and peeled to separate the skin. The skin was chopped into small pieces, homogenized in distilled water (1:5 w/v), and soaked in a 20% NaCl solution prepared in 0.05M Tris–HCl solution for six hours, followed by filtration. The residue was suspended in 0.45M NaCl solution in 0.05 M Tris–HCl solution at pH 7.5 for 48 h. The suspension was filtered, and the filtrate was treated with 0.5M acetic acid for 48 h. The suspension was centrifuged at 4000×*g* for 30 min, and the residue was soaked in 1M NaCl in 0.05M Tris–HCl at pH 7.5 for 12 h, centrifuged, and the pallet of collagen was dried at room temperature and preserved in a refrigerator at 4 °C until usage [19].

## 7. Animals

The white New Zealand albino rabbits (n = 15, weight: 1.2 ± 2 kg, age: 6–9 months) were purchased from the local market. The animals were acclimated to an underground natural environment at 25 ± 2 °C temperature, 50–60% humidity, and 10/14 h light/dark duration for one week and fed three times a day on a controlled diet consisting of a blend of wheat, maize, barley, and peas, and given unrestricted access to fresh water. The animals were sacrificed and properly disposed of in soil at the end of the experiment.

## 8. Induction and treatment of rheumatoid arthritis

The animals were divided into five groups, each consisting of three animals. Four out of five groups were injected in the base of the tail with complete Freund’s adjuvant and collagen emulsion prepared in normal saline (1:1, 250 μL/kg body weight) to induce RA [20]. The injection was repeated three times after one week. The fifth one was left untreated as a control. Three RA groups were treated with ZnO NPs, curcumin, and ZnO NPs-DC (1000 μL/kg body weight) [21]. The dose was repeated three times after one week. The fourth group was left untreated as RA control.

## 9. Blood sampling

Blood samples were collected before and after one week of RA induction and treatment of RA. Blood samples were drawn from the middle vein of the ear using a sterile syringe needle in serum tubes containing a gel and clot activator and centrifuged at 4000×*g* for 20 min [22].

Then, the serum was collected and stored at −20 °C in Eppendorf tubes till analysis. The model setup, blood collection and treatment plan is presented in Table 1.

## 10. Rheumatoid factor test

The RA latex test kit was used for the rheumatoid factor (RF) test [23]. The serum (100 μl was treated with a highly purified human gamma globulin coated on polystyrene latex particles comprising the RA test antigen. The agglutination in the suspension of coated latex particles and serum indicated the positive rheumatoid factor at quantities equivalent to or higher than the sensitivity 8 IU/ml, as identifiable by slide test procedures.

## 11. C reactive protein test

The C-reactive protein (CRP) latex test kit performed the CRP test as a biomarker of RA [24]. The serum (100 μl) was mixed with the CRP latex reagent. The appearance of agglutination indicated the presence of CRP equivalent to or higher than 0.6 mg/dL.

## 12. Oxidative stress

The oxidative stress in the sera was determined regarding malondialdehyde (MDA) production following the previously reported method [25]. The serum (100 μL) was treated with 46 mM thiobarbituric acid solution prepared in 99% glacial acetic acid. The reaction mixture was heated for 35 min and left to cool at room temperature. The absorbance of the MDA-TBA adduct noted at 562 nm was proportional to MDA production and oxidative stress.

## 13. Antioxidant potential

The antioxidant potential of the serum was determined in terms of 2,2-diphenyl-1picrylhydrazyl radical scavenging capacity (DPPH RSC), total antioxidant activity (TAOA), linoleic acid reduction capacity (LARC), superoxide dismutase (SOD) activity, and catalase activity.

The previously reported method determined the DPPH RSC [26]. The serum sample (100 μL) was mixed with 40 mM DPPH solution (3.5 mL) prepared in methanol and kept in darkness for 30 min. Methanol and DPPH solution without serum were taken as blank and control, respectively. The absorbance of the reaction mixture was noted at 517 nm using a spectrophotometer, and DPPH RSC was calculated as follows.


$$\text{DPPH RSC}(\%)={Abs}_{control}-{Abs}_{sample}/{Abs}_{control}\times 100$$

TAOA was determined by phosphomolybdenum assay [27]. The serum (100 μL) was mixed with 28 mM sodium phosphate solution (3 mL), 0.6 M sulfuric acid solution (3 mL), and 4 mM ammonium molybdate solution (3 mL). The reaction mixture was allowed to stand in the water bath for 90 min at 37 °C, cooled to room temperature, and the absorbance was measured at 695 nm using a spectrophotometer against a blank (without reagent). Butylated hydroxytoluene (BHT) equivalent TAOA was calculated using a regression equation derived from the BHT standard curve (R2 = 0.9771).


$$\text{TAOA }(\text{mg}/100\;\text{mL})={Abs}_{sample}/0.3629$$

The ferric thiocyanate approach was used to test the LARC of the sample [27]. The serum (100 μL) was mixed with 2.5% linoleic acid solution (2 mL) prepared in ethanol, 0.05M sodium phosphate buffer (2 mL), and distilled water (2 mL). After incubating at 40 °C in the dark for 24 h, 75% aqueous methanol (10 mL), 20 mM FeCl_3_ solution (1 mL), and 15% ammonium thiocyanate solution (1 mL) were added to the reaction mixture. The reaction mixture without a sample was taken as a control, and the BHT solution was taken as standard. The absorbance was taken at 500 nm against a blank (without FeCl_3_ solution), and the LARC was determined as follows.


LARC (%)=Abscontrol-Abssample/Abscontrol×100

SOD activity was determined in reverse proportion to the absorbance of the superoxide radicalnitro blue tetrazolium (NBT) complex [28]. The serum (100 μL) was mixed with 0.75 mM NBT (100 μL), 3 mM hypoxanthine (100 μL), and 50 mM sodium carbonate buffer (2.4 mL). The reaction was initiated by adding 0.75 mM xanthine oxidase (10 μL). The absorbance was noted at 560 nm, and SOD activity was considered inversely proportional to the absorbance.

Catalase activity was determined in reverse proportion to the formation of potassium dichromate-hydrogen peroxide complex [29]. The serum (100 μL) was mixed with 2M hydrogen peroxide solution (0.4 mL) and 0.01 M phosphate buffer (0.2 mL). An aliquot (1 mL) of the reaction mixture was mixed with dichromate acetic acid solution (2 mL). The reaction mixture was incubated at 37 °C in a water bath for 10 min. The catalase activity was taken inversely proportional to the absorbance of the reaction mixture at 570 nm.

## 14. Statistical analysis

The result of serum MDA content, DPPH RSC, TAOA, LARC, SOD activity, and catalase activity before and after RA induction and treatment were presented as mean ± standard deviation of three replicates. The data was analyzed statistically with a one-way analysis of variance (ANOVA) in statistical software (SPSS version 23) utilizing Turkey’s multiple range test.

## 15. Results and discussion yield and physical characterization of nanoparticles

**Fig. 1:**
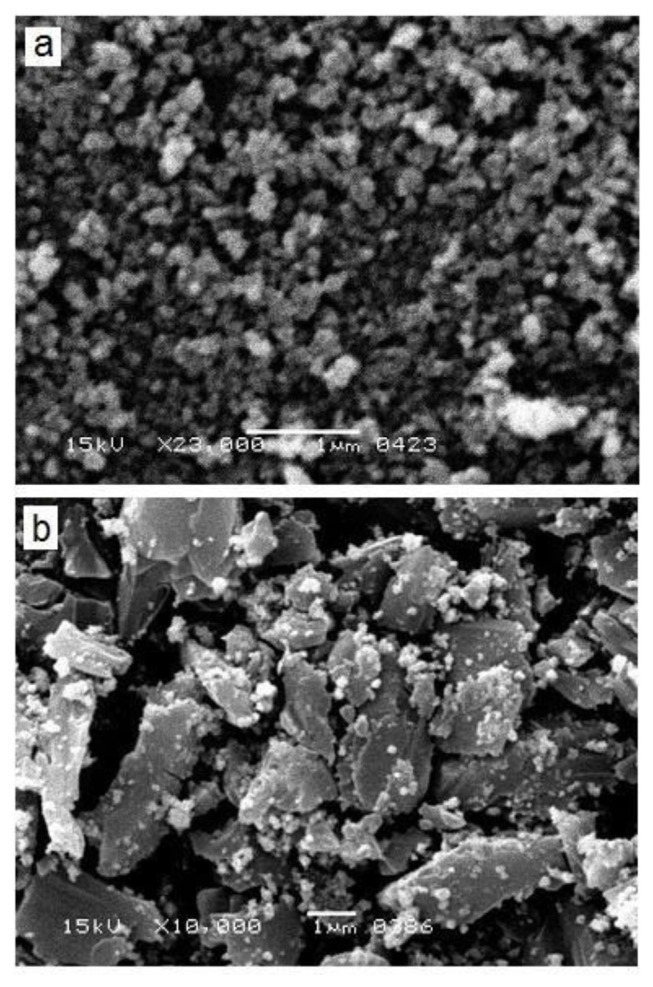
Scanning electron microscopy (SEM) image of a) Zinc oxide nanoparticles (ZnO NPs) and b) Zinc oxide nanoparticles-doped curcumin (ZnONPs-DC).

**Fig. 2:**
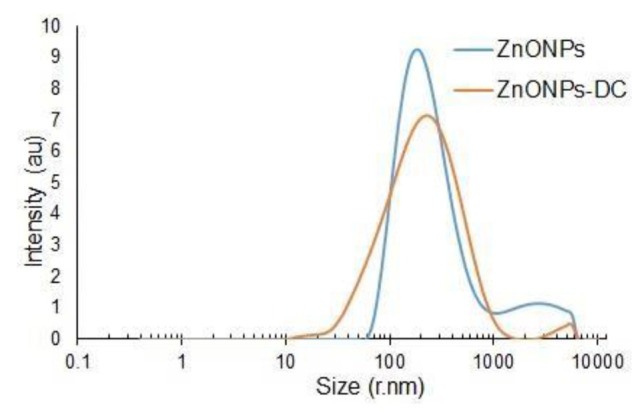
Dynamic Light Scattering (DLS) particle size distribution of Zinc oxide nanoparticles (ZnO NPs) and Zinc oxide nanoparticles-doped curcumin (ZnONPs-DC)

**Fig. 3:**
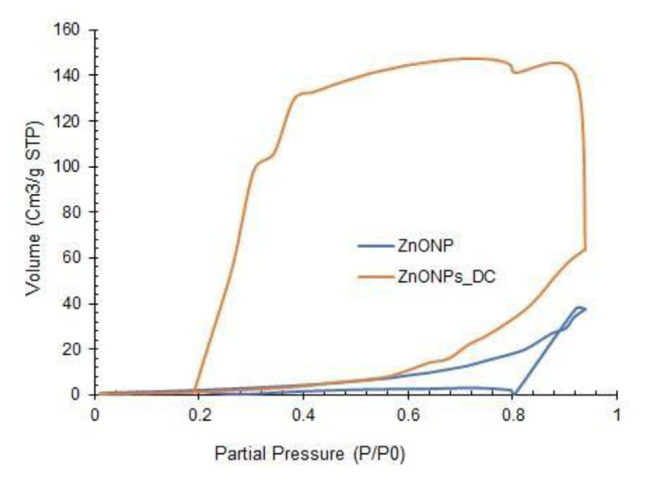
Brunauer–Emmett–Teller (BET) adsorption/desorption isotherms of Zinc oxide nanoparticles (ZnO NPs) and Zinc oxide nanoparticles-doped curcumin (ZnONPs-DC).

ZnONPs, curcumin, and ZnONPs-DC yielded 7.5 ± 1.25, 3 ± 0.5 g, and 4 ± 1.5 g, respectively. Fig. 1 represents the results of SEM analysis of ZnONPs and ZnONPs-DC. The SEM images showed irregularly shaped nano-sized particles of ZnO. Doping of ZnONPs on curcumin resulted in the adsorption of small particles on the clusters of curcumin, showing the amorphous morphology of ZnONPs-DC. The results of particle size distribution of ZnONPs and ZnONPs-DC as analyzed by DLS are presented in Fig. 2. The ZnONPs particles of size 60–190 nm showed increasing light scattering intensity, which decreased in larger particles due to reduced surface area relative to their volume. The ZnONPs-DC showed a particle size distribution in the 30–255 nm range with relatively lower intensity. Fig. 3 represents the BET patterns of N_2_ adsorption/desorption on the surface of ZnONPs and ZnONPs-DC. The ZnO NPs showed maximum gas adsorption at a partial pressure of 0.9 P/P0 and formed a monolayer to interact with gas molecules. The desorption curve showed a fast desorption of gas at 0.8 P/P_0_. The ZnONPs-DC showed relatively similar adsorption but different desorption patterns with slow desorption of N_2_ up to 0.2 P/P_0,_ indicating the multilayer adsorption of ZnO NPs-DC with increased pressure. The slow desorption of adsorbed molecules with decreasing pressure may result from the strong interaction of gas molecules with the nanoparticle’s surface.

## 16. Physical observations after induction and treatment of rheumatoid arthritis

After the induction of RA, the rabbits show signs of reduced activity, tiredness, stiffness in their limbs, aggressive behavior and restricted movement. Along with a decreased appetite and weight loss, they exhibit indicators of physical pain and discomfort. After the nanoparticle treatment, every rabbit survived with recovery of the above mentioned physical changes.

## 17. Rheumatoid factor and C-reactive protein

The results of RF and CRP screening of the control and the study groups are presented in Table 2. The RF and CRP were negative, with no serum agglutination in all groups before RA induction (RF: <8 IU/mL, CRP: <0.6 mg/dL). RF and CRP were found positive with visible agglutination after RA induction (RF > 8 IU/mL, CRP >0.6 mg/dL), indicating the onset of RA. The ZnONPs, curcumin, and ZnO NPs-DC administration to the study groups resulted in a negative RF and CRP, showing the effectiveness of the suggested treatments in reducing inflammation. Oxidative stress markers of RA.

## 18. Oxidative stress and antioxidant potential

**Fig. 4:**
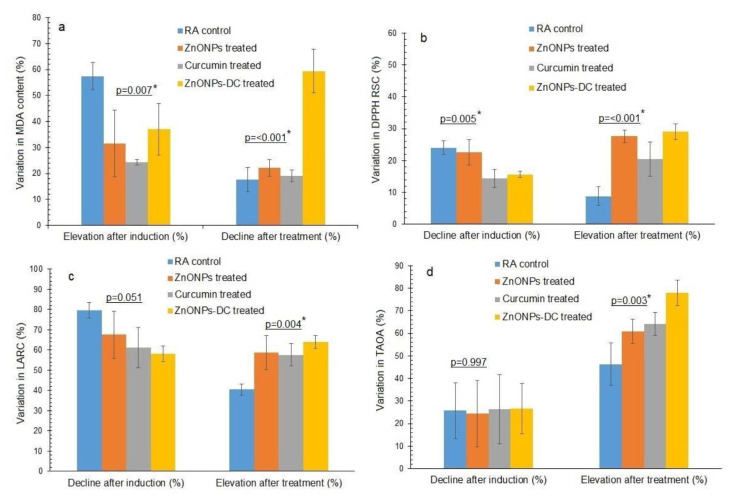
Variation in parameters of oxidative stress and antioxidant potential of RA control and different study groups before and after induction and treatment of rheumatoid arthritis a) Malondialdehyde (MDA), b) 2, 2 diphenyl-1-picrylhydrazyl radical scavenging capacity (DPPH RSC), c) Linoleic acid reduction capacity (LARC), d) Total antioxidant activity (TAOA). RA: Rheumatoid arthritis, ZnONPs: Zinc oxide nanoparticles, ZnONPs-DC: Zinc oxide nanoparticles doped curcumin. *The p-values labelled with the star are statistically different at a 95% confidence level (p ≤ 0.05) by one-way analysis of variance (ANOVA) using Duncan’s multiple range test (SPSS version 23).

The MDA content, DPPH RSC, LARC, TAOA, SOD activity, and catalase activity of RA control, ZnONPs, curcumin, and ZnO NPs-DC treated groups before and after RA induction and treatment are presented in Table 3. The percentage variation in these parameters before and after RA induction and therapy is illustrated in Fig. 4a–d. Before RA induction, the oxidative stress determined as MDA content, in terms of Abs at 562 nm, of the control, ZnONPs, curcumin, and ZnONPs-DC were 0.17 ± 0.014, 0.23 ± 0.04, 0.24 ± 0.21, and 0.14 ± 0.04 respectively. After induction, the MDA levels of the control group, ZnONPs, curcumin, and ZnONPs-DC were increased to 0.40 ± 0.05, 0.33 ± 0.06, 0.31 ± 0.28, and 0.23 ± 0.08 Abs at 562 nm, with a percentage increase of 57.50 ± 5.17, 31.56 ± 12.85, 24.31 ± 0.98, and 37.06 ± 9.89% respectively. The nanoparticles treatment reduced the MDA content of RA control, ZnONPs, Curcumin, and ZnONPs-DC treated groups to 0.33 ± 0.04, 0.26 ± 0.03, 0.25 ± 0.21 and 0.09 ± 0.03 Abs at 562 nm with percentage decline of 17.60 ± 4.74, 22.06 ± 3.22, 18.98 ± 8.43, and 59.42 ± 8.4%, respectively (Fig. 4a). The RA control and all the treatment groups showed a statistically significant difference in percentage induction (p = 0.007) and recovery (p = 0.00).

Before induction, the DPPH RSC of RA control, ZnONPs, curcumin, and ZnONPs-DC were 17.27 ± 24.64, 16.57 ± 4.05, 10.51 ± 2.17, and 15.81 ± 6.37% respectively. Before induction, the LARC of RA control, ZnONPs, curcumin, and ZnONPs-DC were 70.004 ± 3.71, 72.24 ± 10.21, 75.01 ± 7.14, and 61.48 ± 5.36%, respectively, and TAOA, in terms of mg BHT eqv/dL, of RA control, ZnONPs, curcumin, and ZnO NPs-DC were 0.304 ± 0.07, 0.177 ± 0.03, 0.21 ± 0.02, and 0.30 ± 0.06 respectively. After induction of RA, the DPPH RSC of RA control, ZnONPs, curcumin, and ZnONPs-DC was reduced to 13.05 ± 3.20, 12.81 ± 3.14, 9.03 ± 2.13, and 13.36 ± 5.46 % with a percentage decrease of 24.05 ± 2.11, 22.62 ± 3.99, 14.41 ± 2.82, and 15.62 ± 0.97% respectively (Fig. 4b) and the LARC of RA control, ZnONPs, curcumin, and ZnONPs-DC was decreased to 14.16 ± 2.07, 24.21 ± 11.42, 28.97 ± 7.07, and 25.82 ± 4.63% with a percentage decrease of 79.65 ± 3.92, 67.51 ± 11.70, 61.20 ± 10.11, and 58.22 ± 3.81% respectively (Fig. 4c). The TAOA of RA control, ZnONPs, curcumin, and ZnONPs-DC was reduced to 0.23 ± 0.07, 0.13 ± 0.04, 0.15 ± 0.02, and 0.22 ± 0.07 mg BHT eqv/dL with the percentage decrease of 25.72 ± 12.37, 24.48 ± 14.74, 26.32 ± 15.34, and 26.58 ± 11.11% respectively (Fig. 4d). The nanoparticles treatment increased the DPPH RSC of RA control, curcumin, ZnONPs, and ZnONPs-DC to 14.55 ± 4.03, 17.68 ± 4.16, 11.37 ± 2.50, and 18.66 ± 6.99% with a percentage increase of 8.83 ± 2.88, 27.61 ± 1.95, 20.48 ± 5.43, and 28.98 ± 2.43% respectively and LARC of RA control, ZnONPs, curcumin, and ZnONPs-DC was increased to 23.71 ± 2.54, 57.24 ± 20.48, 68.35 ± 15.02 and 71.48 ± 6.83% with a percentage increase of 40.46 ± 2.67, 58.83 ± 8.50, 57.61 ± 5.54, and 64.05 ± 3.20% respectively. The TAOA of control, ZnONPs, curcumin, and ZnONPs-DC was increased to 0.43 ± 0.17, 0.34 ± 0.06, 0.43 ± 0.09, and 1.03 ± 0.25 (mg BHT eqv/dL) with a percentage increase of 46.38 ± 9.48, 60.96 ± 5.36, 64.11 ± 5.17, and 78.02 ± 5.62% respectively. For DPPH RSC, the RA control and all the treatment groups showed a statistically significant difference in percentage induction (p = 0.005) and recovery (p = 0.00). For LARC, the RA control and all the treatment groups showed a statistically significant difference in percentage induction (p = 0.05) and recovery (p = 0.04). For TAOA, the control and all the treatment groups showed a statistically significant difference in percentage recovery (p = 0.003).

**Fig. 5:**
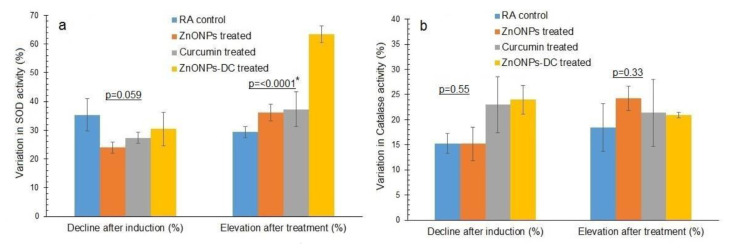
Variation in the activity of antioxidant enzymes of RA control and different study groups before and after induction and treatment of rheumatoid arthritis a) Superoxide dismutase (SOD) activity, b) Catalase activity. RA: Rheumatoid arthritis, ZnONPs: Zinc oxide nanoparticles, ZnONPs-DC: Zinc oxide nanoparticles doped curcumin. *The p-values labelled with the star are statistically different at a 95% confidence level (p ≤ 0.05) by one-way analysis of variance (ANOVA) using Duncan’s multiple range test (SPSS version 23).

Before RA induction, the absorbance taken in reverse proportion to SOD activity of RA control, ZnONPs, curcumin, and ZnONPs-DC was 0.04 ± 0.004, 0.03 ± 0.001, 0.04 ± 0.02, and 0.05 ± 0.02 respectively (Abs at 560 nm). The absorbance taken in reverse proportion to catalase activity of the RA control, ZnONPs, curcumin, and ZnONPs-DC was 0.46 ± 0.005, 0.43 ± 0.02, 0.45 ± 0.04 and 0.44 ± 0.02 (Abs at 570 nm) respectively. After induction of RA, the SOD absorbance of RA control, ZnONPs, curcumin, and ZnONPs-DC was increased to 0.06 ± 0.002, 0.04 ± 0.002, 0.05 ± 0.03, and 0.07 ± 0.02 (Abs at 560 nm) with a percentage decrease of SOD activity as 35.39 ± 5.68, 24.04 ± 1.94, 27.39 ± 1.88, and 30.48 ± 5.87% respectively (Fig. 5a) and the catalase activity absorbance of the RA control, ZnONPs, curcumin and ZnONPs-DC was increased to 0.471 ± 0.01, 0.48 ± 0.01, 0.47 ± 0.01, and 0.47 ± 0.04 (Abs at 570 nm), with a percentage decline in catalase activity as 5.18 ± 3.80, 8.11 ± 4.07, 4.96 ± 0.43, and 6.59 ± 1.64% respectively (Fig. 5b). After treatment of RA, the absorbance for measuring SOD activity of the RA control, ZnONPs, curcumin, and ZnONPs-DC was reduced to 0.04 ± 0.001, 0.03 ± 0.001, 0.03 ± 0.014, and 0.02 ± 0.004 (Abs at 560 nm) with a percentage increase in SOD activity to 29.43 ± 1.94, 36.06 ± 2.91, 37.33 ± 6.08, and 63.43 ± 2.93% respectively and catalase activity absorbance of the RA control, ZnONPs, curcumin and ZnONPs-DC was decreased to 0.39 ± 0.011, 0.36 ± 0.017, 0.38 ± 0.02 and 0.351 ± 0.023 (Abs at 570 nm) with a percentage increase in catalase activity up to 19.07 ± 3.71, 22.14 ± 2.21, 17.39 ± 10.1, and 25.73 ± 1.64% respectively. For SOD activity, the RA control and all treatment groups showed a statistically significant difference in percentage recovery (p = 0.00).

The pathophysiology of RA has been studied using rabbit adjuvant-induced arthritis as an experimental model. Zinc oxide nanoparticles have been proven to have anti-arthritic action by decreasing inflammation and oxidative stress [14]. Curcumin also has shown curative effects in RA therapy by lowering inflammation and clinical symptoms [11].

This study investigated the prospective therapeutic effects of combining Curcumin and ZnONPs on alleviating RA. The physical characterization of synthesized ZnO NPs, CNPs, and ZnO NPs-DC showed characteristic morphological and structural features. The SEM analysis showed the irregular shape of ZnONPs in the form of agglomerates, and these results were comparable to those reported earlier for ZnONPs [30]. Our findings demonstrated the amorphous morphology of ZnONPs-DC, which could help improve the solubility, bioavailability, and therapeutic effectiveness of water-insoluble medications. The DLS technique was used to measure the size of NPs based on the intensity of dispersed light. The compatibility of NPs with biological systems can be evaluated by evaluating their size. Relatively large NPs may have trouble overcoming natural barriers. Hence, too small NPs may be quickly eliminated by the immune system. The effectiveness of their targeting and cellular uptake is also impacted by NPs size [31]. BET analysis was performed to evaluate the adsorption properties of synthesized NPs. In our results, the adsorption capacity of ZnONPs and ZnONPs-DC showed significance in preventing bioavailability hurdles and improving drug administration [32].

RA is characterized by autoimmunity, inflammation, and joint destruction. The autoimmunity includes the autoantibodies such as RF, CRP, and anti-CCP that are specifically produced for IgG. Previous studies on curcumin therapy to treat RA also suggested it is a safe agent for lowering RF and CRP and improving disease symptoms [33]. In our study, after induction of RA, positive RF and CRP with observed agglutination of serum samples showed the successful induction of RA in rabbits. After treating arthritic rabbits with different treatment groups, surprisingly, all the treatment groups showed negative RF and CRP and provided a remarkable therapeutic effect in combating disease symptoms.

Before the induction of RA, a slight variation in serum MDA levels among the four treatment groups can be related to inheritance, physiologic variations, pre-existing medical problems, age, and weight. Increased oxidative stress due to ongoing inflammation and imbalanced antioxidant defence systems is responsible for increased MDA levels after the onset of rheumatoid arthritis [34]. Our study showed a statistically significant change in serum MDA levels before and after induction and after treatment of RA. In our research, ZnO NPsDC treatment showed the highest recovery by decreasing serum MDA levels, and it highlights the possible beneficial effects of using a combination of ZnONPs and curcumin in RA management due to their antioxidant, immunomodulatory, and anti-inflammatory properties. Curcumin and ZnONPs also reduced serum MDA content due to their potential to reduce lipid peroxidation produced by hydrogen peroxide.

After the induction of RA, the serum level of DPPH RSC, LARC, and TAOA decreased. This decrease might be attributed to increased antioxidant consumption because RA is described by continuous inflammatory processes and enhanced oxidative stress [35]. The serum’s ability to scavenge DPPH radicals was highly increased by treating arthritic rabbits with ZnO NPs-DC due to their antioxidant and anti-inflammatory properties. These agents can be safe to be used in clinical practice to lower RA symptoms. All other treatment groups also improved DPPH RSC. In LARC, ZnO NPs-DC displayed exceptional pharmacological effects due to its efficacy in strengthening the antioxidant defense mechanisms. Unsaturated fats in cellular membranes are the main targets for free radical-caused chain reactions because they are sensitive to oxidative damage. The process is often controlled by endogenous antioxidant molecules that decrease unsaturated fatty acids to minimize lipid peroxidation [36]. ZnO NPsDC treatment group showed a considerable increase in TAOA levels, showing the highest recovery percentage by creating antioxidants to combat illness. Levels of TAOA were also considerably raised by ZnONPs and curcumin.

The decline in SOD activity after RA induction may be due to the inactivation of the enzyme in response to increased oxidative stress. Antioxidants, such as superoxide dismutase and α-tocopherol, work as inflammation inhibitors in experimentally induced arthritis [37]. ZnO NPs-DC showed a remarkable pharmacological impact in improving SOD activity. It may be due to curcumin’s ability to inhibit inflammatory pathways by raising SOD and GSH levels. Inflammation, antioxidant capacity, and signaling mechanisms downstream of SOD are all known to be affected by curcumin. Our results emphasized combining ZnONPs and curcumin to manage the RA symptoms.

Our study showed lower catalase activity in the serum of RA rabbits. Catalase prevents ROS-mediated damage by converting hydrogen peroxide to water and oxygen. In our RA patients, catalase activity is significantly decreased compared to control. Lower catalase activity may be due to the interaction of catalase with hydrogen peroxide. Lowered activities of their enzymes may lead to the conversion of hydrogen peroxide to hydroxyl radical by iron released from the hemoglobin of lysed erythrocytes [38]. All the treatments resulted in an elevation in catalase activity. However, ZnONPs-DC showed the highest recovery percentage by increasing catalase activity. It may be due to the combined anti-inflammatory and antiarthritic properties of ZnONPs-DC, and it proved itself a safe treatment for the management of RA.

## 19. Conclusions

In conclusion, it was found that CFA and collagen type-II emulsion resulted decreased physical activity, inflammation in joints, positive RF and CRP, increased oxidative stress, and decreased antioxidant levels indicating the induction of RA in rabbits. The ZnONPs-DC treatment resulted in recovery of RA observed as improvement in physical activity, decreased inflammation, negative RF and CRP, improved antioxidant status in terms of decreased serum MDA levels and enhanced DPPH RSC, TAOA, LARC, and SOD activities. The logical interpretations conclude that metal-based nanocomposite therapy effectively treats RA. In order to implement these nanocomposites into clinical practice for treating RA, additional research is required to clarify intricate mechanisms.

## Figures and Tables

**Table 1:** The model setup for induction of rheumatoid arthritis, treatment, and blood sampling.

Study group	RA induction			Treatment		
	
	CFA-Collagen II treatment	No of dose/dose interval	Sampling	NPs and curcumin treatment	No of dose/dose interval	Blood sampling
Normal control	Untreated	0	21st day of experiment	Untreated	0	42nd day of experiment
RA control	250 μl/kg bw	3/7 days	7 days after last dose	Untreated	0	21 days after RA induction
ZnO NPs treated	250 μl/kg bw	3/7 days	7 days after last dose	1 ml/kg bw	3/7 days	7 days after last dose
Curcumin treated	250 μl/kg bw	3/7 days	7 days after last dose	1 ml/kg bw	3/7 days	7 days after last dose
ZnO NPs-DC treated	250 μl/kg bw	3/7 days	7 days after last dose	1 ml/kg bw	3/7 days	7 days after last dose

* bw: body weight, CFA: Complete Freunds adjuvant, RA: Rheumatoid arthritis, ZnONPs:Zinc oxide nanoparticles, ZnONPs-DC: Zinc oxide nanoparticles doped curcumin.

**Table 2:** Biological parameters of the control and study groups before and after induction and treatment of Rheumatoid arthritis.

Groups	Before induction	After induction	After treatment
Rheumatoid factor (IU/mL)			
Normal control	<8	<8	<8
RA control	<8	≥8	<8
ZnONPs	<8	≥8	<8
Curcumin	<8	≥8	<8
ZnONPs-DC	<8	≥8	<8
C-Reactive Protein level (mg/L)			
Normal control	<6	<6	<6
RA control	<6	≥6	<6
ZnONPs	<6	≥6	<6
Curcumin	<6	≥6	<6
ZnONPs-DC	<6	≥6	<6

* RA: Rheumatoid arthritis, ZnONPs: Zinc oxide nanoparticles, ZnONPs-DC: Zinc oxide nanoparticles-doped curcumin.

**Table 3:** Oxidative stress and antioxidant potential of control and study groups before and after induction and treatment of Rheumatoid arthritis.

Groups	Before RA After RA After Curcumin Induction induction induction and NPs treatment (%)				Recovery (%)
Malondialdehyde content (Absorbance at 562 nm)					
Normal Control	0.194 ± 0.103^a^	0.201 ± 0.09^a^	0.196 ± 0.105^a^		
RA Control	0.17 ± 0.014^a^	0.40 ± 0.05^a^	0.33 ± 0.04^a^	57.50 ± 5.17^a^	17.60 ± 4.74^a^
ZnO NPs	0.23 ± 0.04^a^	0.33 ± 0.06^a^	0.26 ± 0.03^a^	31.56 ± 12.85^a^	22.06 ± 3.22^a^
Curcumin	0.24 ± 0.21^a^	0.31 ± 0.28^a^	0.25 ± 0.21^a^	24.31 ± 0.98^a^,^b^	18.98 ± 8.43^a^
ZnONPs-DC	0.14 ± 0.04^a^	0.23 ± 0.08^a^	0.09 ± 0.03^a^	37.06 ± 9.89^b^	59.42 ± 8.4^b^
p-value	0.694	0.612	0.150	0.007	<0.001
2, 2 diphenyl-1-picrylhydrazyl radical scavenging capacity (%)					
Normal control	15.04 ± 4.78^a^	16.02 ± 4.54^a^	14.72 ± 3.94^a^		
RA Control	17.27 ± 4.64^a^	13.05 ± 3.20^a^	14.55 ± 4.03^a^	24.05 ± 2.11^a^	8.83 ± 2.88^a^
ZnO NPs	16.57 ± 4.05^a^	12.81 ± 3.14^a^	17.68 ± 4.16^a^	22.62 ± 3.99^a^,^b^	27.61 ± 1.95^b^
Curcumin	10.51 ± 2.17^a^	9.03 ± 2.13^a^	11.37 ± 2.50^a^	14.41 ± 2.82^b^,^c^	20.48 ± 5.43^b^
ZnONPs-DC	15.81 ± 6.37^a^	13.36 ± 5.46^a^	18.66 ± 6.99^a^	15.62 ± 0.97^c^	28.98 ± 2.43^b^
p-value	0.322	0.479	0.293	0.005	<0.001
Linoleic acid reduction capacity (%)					
Normal Control	69.68 ± 7.98^a^	70.34 ± 6.65^a^	69.97 ± 7.87^a^		
RA Control	70.004 ± 3.71^a^	14.16 ± 2.07^a^	23.71 ± 2.54^a^	79.65 ± 3.92^a^	40.46 ± 2.67^a^
ZnO NPs	72.24 ± 10.21^a^	24.21 ± 11.42^a^	57.24 ± 20.48^a^,^b^	67.51 ± 11.70^a^	58.83 ± 8.50^b^
Curcumin	75.01 ± 7.14^a^	28.97 ± 7.07^a^	68.35 ± 15.02^b^	61.20 ± 10.11^a^	57.61 ± 5.54^b^
ZnONPs-DC	61.48 ± 5.36^a^	25.82 ± 4.63^a^	71.48 ± 6.83^b^	58.22 ± 3.81^a^	64.05 ± 3.20^b^
p-value	0.183	0.145	0.008	0.051	0.004
Total antioxidant activity (mg BHT Eqv/dL)					
Normal control	0.249 ± 0.07^a^	0.264 ± 0.05^a^	0.251 ± 0.06^a^		
RA Control	0.304 ± 0.07^a^	0.23 ± 0.07^a^	0.43 ± 0.17^a^	25.72 ± 12.37^a^	46.38 ± 9.48^a^
ZnO NPs	0.177 ± 0.03^a^	0.13 ± 0.04^a^	0.34 ± 0.06^a^	24.48 ± 14.74^a^	60.96 ± 5.36^a^,^b^
Curcumin	0.21 ± 0.02^a^	0.15 ± 0.02^a^	0.43 ± 0.09^a^	26.32 ± 15.34^a^	64.11 ± 5.17^b^
ZnONPs-DC	0.30 ± 0.06^a^	0.22 ± 0.07^a^	1.03 ± 0.25^b^	26.58 ± 11.11^a^	78.02 ± 5.62^b^
p-value	0.039	0.177	0.003	0.997	0.003
Superoxide dismutase activity (Absorbance at 560 nm)					
Normal control	0.039 ± 0.01^a^	0.041 ± 0.008^a^	0.043 ± 0.007^a^		
RA Control	0.04 ± 0.004^a^	0.06 ± 0.002^a^	0.04 ± 0.001^a^	35.39 ± 5.68^a^	29.43 ± 1.94^a^
ZnO NPs	0.03 ± 0.001^a^	0.04 ± 0.002^a^	0.03 ± 0.001^a^	24.04 ± 1.94^a^,^b^	36.06 ± 2.91^a^
Curcumin	0.04 ± 0.02^a^	0.05 ± 0.03^a^	0.03 ± 0.014^a^	27.39 ± 1.88^a^,^b^	37.33 ± 6.08^a^
ZnONPs-DC	0.05 ± 0.02^a^	0.07 ± 0.02^a^	0.02 ± 0.004^a^	30.48 ± 5.87^b^	63.43 ± 2.93^b^
p-value	0.311	0.295	0.157	0.059	<0.0001
Catalase activity (Absorbance at 570 nm)					
Normal control	0.453 ± 0.028^a^	0.471 ± 0.01^a^	0.459 ± 0.02^a^		
RA Control	0.46 ± 0.005^a^	0.48 ± 0.01^a^	0.39 ± 0.011^a^	5.18 ± 3.80^a^	19.07 ± 3.71^a^
ZnO NPs	0.43 ± 0.02^a^	0.47 ± 0.01^a^	0.36 ± 0.017^a^	8.11 ± 4.07^a^	22.14 ± 2.21^a^
Curcumin	0.45 ± 0.04^a^	0.47 ± 0.04^a^	0.38 ± 0.02^a^	4.96 ± 0.43^a^	17.39 ± 10.1^a^
ZnONPs-DC	0.44 ± 0.02^a^	0.47 ± 0.02^a^	0.351 ± 0.023^a^	6.59 ± 1.64^a^	25.73 ± 1.64^a^
p-value	0.66	0.91	0.12	0.55	0.33

a The results are represented as means ± SD. The means labelled with the same alphabets in the same column are statistically similar at a 95% confidence level (p ≤ 0.05) by one-way analysis of variance (ANOVA) using Duncan’s multiple range test (SPSS version 23).

b RA: Rheumatoid arthritis, ZnONPs: Zinc oxide nanoparticles, ZnONPs-DC: Zinc oxide nanoparticles-doped curcumin.

## Data Availability

All the data associated with this study are presented in Tables and Figs.

## List of Abbreviations

BET: Brunauer-Emmet-Teller
CRP: C-reactive protein
DLS: Dynamic light scattering
DMARDs: Disease-modifying anti-rheumatic drugs
DPPH RSC: 2,2-diphenyl-1picrylhydrazyl radical scavenging capacity
GC: Glucocorticoids
LARC: Linoleic acid reduction capacity
MDA: Malondialdehyde
RA: Rheumatoid arthritis
RF: Rheumatoid factor
ROS: Reactive oxygen species
SEM: Scanning electron microscopy
SOD: Superoxide dismutase
TAOA: Total antioxidant activity
ZnONPs: Zinc oxide nanoparticles
ZnONPsDC: Zinc oxide nanoparticles-doped curcumin
